# Melioidosis in the Philippines

**DOI:** 10.3390/tropicalmed3030099

**Published:** 2018-09-05

**Authors:** Peter Franz M. San Martin, Joseph C. Chua, Ralph Louie P. Bautista, Jennifer M. Nailes, Mario M. Panaligan, David A. B. Dance

**Affiliations:** 1 Department of Physiology, College of Medicine, University of the East—Ramon Magsaysay Memorial Medical Center Inc., Aurora Boulevard, Quezon City 1113, Philippines; joseph_c_chua@yahoo.com (J.C.C.); rlpbautista@gmail.com (R.L.P.B.); 2 Department of Preventive and Community Medicine, College of Medicine, University of the East—Ramon Magsaysay Memorial Medical Center Inc., Aurora Boulevard, Quezon City 1113, Philippines; jmnailes@uerm.edu.ph; 3 Department of Medicine, College of Medicine, University of the East—Ramon Magsaysay Memorial Medical Center Inc., Aurora Boulevard, Quezon City 1113, Philippines; mmpanaligan@gmail.com; 4 Lao-Oxford-Mahosot Hospital-Wellcome Trust Research Unit, Microbiology Laboratory, Mahosot Hospital, Vientiane, Laos; David.d@tropmedres.ac; 5 Centre for Tropical Medicine and Global Health, Nuffield Department of Clinical Medicine, Old Road Campus, University of Oxford, Oxford OX3 7FZ, UK; 6 Faculty of Infectious and Tropical Diseases, London School of Hygiene and Tropical Medicine, London WC1E 7HT, UK

**Keywords:** melioidosis, Philippines, *Burkholderia pseudomallei*

## Abstract

The first documented case of melioidosis in the Philippines occurred in 1948. Since then, there have been sporadic reports in the literature about travelers diagnosed with melioidosis after returning from the Philippines. Indigenous cases, however, have been documented rarely, and under-reporting is highly likely. This review collated all Philippine cases of melioidosis published internationally and locally, as well as unpublished case series and reports from different tertiary hospitals in the Philippines. In total, 25 papers and 41 cases were identified. Among these, 23 were indigenous cases (of which 20 have not been previously reported in the literature). The most common co-morbidity present was diabetes mellitus, and the most common presentations were pulmonary and soft tissue infections. Most of the cases received ceftazidime during the intensive phase, while trimethoprim-sulfamethoxazole was given during the eradication phase. The known mortality rate was 14.6%, while 4.9% of all cases were reported to have had recurrence. The true burden of melioidosis in the country is not well defined. A lack of awareness among clinicians, a dearth of adequate laboratories, and the absence of a surveillance system for the disease are major challenges in determining the magnitude of the problem.

## 1. Introduction and History of Melioidosis in the Philippines

Melioidosis, or Whitmore’s disease, is a potentially lethal infection caused by the Gram-negative bacterium *Burkholderia pseudomallei*. Its clinical presentation is so diverse that the diagnosis relies heavily on laboratory culture.

During the past few decades, melioidosis has emerged as an important public health concern, especially in the South East Asian (SEA) region and northern Australia [1]. The increase in global interest in the disease was a consequence of the rising number of reported cases, both within endemic areas and among travelers, the occurrence of cases in areas not previously known to be endemic; and also, its potential use as an agent of bioterrorism.

After the disease was first described by Alfred Whitmore and C.S. Krishnaswami in 1912 [1,2,3], initially the majority of cases reported were from the SEA region. Reviews of the global distribution of the disease showed that cases have been found in the tropical and subtropical regions, mainly between 20° north and 20° south of the equator—which includes the Philippines [2,4]. A recent modelling study suggested that there could be as many as 9000 cases and 4500 deaths, due to melioidosis occurring in the Philippines each year [5]. Certainly, nowhere near that number are being diagnosed, so how likely is this to be true?

The first documented case of human melioidosis in the Philippines occurred in 1948 in a 25-year-old American soldier who presented with weight loss and symptoms of pneumonia. Diagnosis was established through guinea pig inoculation of sputum and lymph node pus [6]. This was not long after the disease had first been recognized in countries like Vietnam [7] and Indonesia [8], and before the first indigenous case was described in Thailand [9]. However, in contrast to other SEA countries, there has been little development with regards to defining the epidemiology of the disease in the Philippines. A further case in an American soldier was reported in 1957, although his illness had started many years earlier, while he was stationed in the Philippines [10]. Sporadic cases were reported thereafter in people who had either transited or stayed in the country. Some of these individuals also had a history of travel to other parts of SEA, hence the evidence that the infection was acquired in the Philippines was inconclusive [2]. However, in others, such as the first case diagnosed in Taiwan in 1985, there was strong circumstantial evidence that infection was acquired in the Philippines during a near-drowning incident in a river near Manila [11]. 

Cases have not been limited to humans: For example, an isolate from a horse from the Philippines was included in a taxonomic study published in 1956, although no further clinical details are available about this case [12]. Then in 1992, a number of primates imported from the Philippines to the United Kingdom were involved in an outbreak of melioidosis [13]. However, there have been no other cases among animals from the Philippines reported in the literature thereafter.

Indigenous cases, meaning those with no apparent travel history to other endemic areas, and who were diagnosed and treated in the Philippines, had never been reported in the literature until 2002, when a 49-year-old farmer from Bulacan in the Philippines presented with pain and swelling of the left shoulder and was found to have melioidosis after *Burkholderia pseudomallei* was isolated from blood [14]. The second indigenous case, and the first case published in an international journal, was reported as recently as 2016 [15]. The patient was a farmer from Isabela with no history of travel outside the country, who was diagnosed and treated in a hospital in Metro Manila. 

The preponderance of reports of melioidosis among travelers from the Philippines, and the paucity of indigenous cases, leads us to suspect that the disease is being grossly under-reported in the country. This article describes the evidence for, and distribution of, melioidosis in the Philippines by reviewing reports published in international and local publications and unpublished case reports that were obtained from tertiary hospitals and local subspecialty organizations. These cases were examined to determine common patient profiles, disease presentations, modes of diagnosis, treatment received, and outcomes.

## 2. Materials and Methods

### 2.1. Review of Publications

Published reports citing cases of culture-confirmed melioidosis from the Philippines were collected. The melioidosis website (www.melioidosis.info) served as the main starting point for the search, as it already contains a database of published case reports by country. In addition, PubMed and Google Scholar were searched, using various combinations of keywords (and MESH terms, where applicable) such as Philippines, Filipino, Southeast Asia, melioidosis, Whitmore’s disease, *Burkholderia pseudomallei*, and *Pseudomonas pseudomallei*. The bibliography of each reference was searched for further cases. Finally, the personal EndNote database of one of us (DABD) was searched for references that had been missed. From each reference, relevant information about each case was extracted, such as age, sex, co-morbidities, risk factors, type of infection, mode of diagnosis, treatment received, outcome, country where diagnosed, and year of diagnosis.

### 2.2. Collection of Locally Published and Unpublished Cases from the Philippines

Local medical journals and university papers available in the National Library of the Philippines, different universities, and hospitals were searched for any mention of local cases of melioidosis. Inquiries regarding cases of melioidosis were made to the Philippine Society for Microbiology and Infectious Diseases, the Research Institute for Tropical Medicine, and other medical centers. Consent to use the data was obtained from the authors of unpublished case series and reports that were collected.

## 3. Results and Discussion

A total of 25 articles, either single case reports or case series, describing culture-confirmed melioidosis in the Philippines were collected (Table 1 and Table 2). Of these 25 papers, only 11 were listed in the melioidosis.org database—although those not included had either been published or presented at international conferences recently (between 2015 and 2017). This also included four unpublished local reports which were obtained from different tertiary hospitals in Metro Manila. Only one of these reports related to a case of melioidosis in an animal [13].

A total of 41 human cases was included in these articles, with 18 cases involving travelers and 23 indigenous cases. Among the indigenous cases, 20 were from the four previously unpublished reports. Of these four unpublished papers, two were case series derived from a 3- to 5-year chart review of patients that were culture-positive for *B. pseudomallei* from their respective institution, and the other two were single case reports. Diagnosis was established via culture-based technique and VITEK among indigenous cases. No additional molecular confirmatory tests were performed for these specimens.

**Figure 1:**
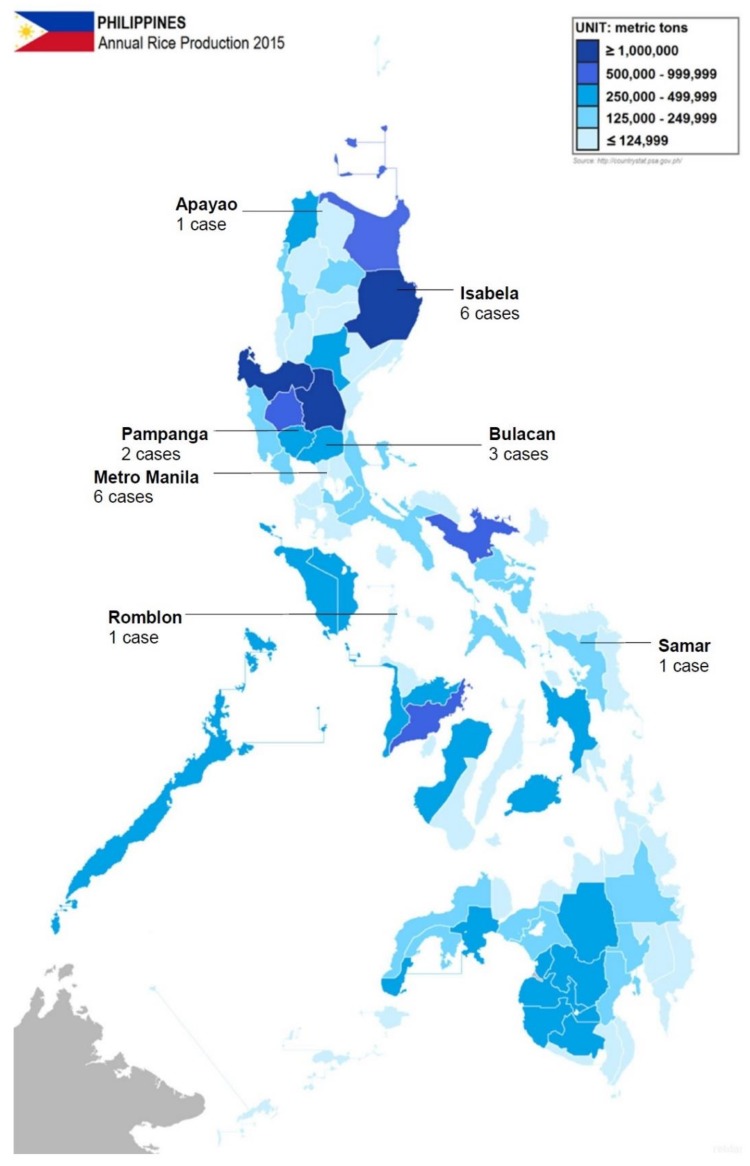
Distribution of indigenous cases of melioidosis in the Philippines (reported from 2002 to 2016) and the amount of rice production per province. Original image imported from: https://commons.wikimedia.org/wiki/File:Philippine_provinces_Annual_Rice_Production_2015.png.

The patients were between the ages of 21 and 82 years, with a mean of 50.2 years (Table 3). The patients were predominantly (85.4%) male. This is consistent with the epidemiologic data from some other endemic areas [16,17,18]. Data on risk factors were limited, but only six patients were known to be working in the agricultural sector. Geographical data were available for 20 indigenous cases, where 11 were from areas with moderate to high rice production in the Philippines (Isabela, Pampanga, and Bulacan). Figure 1 shows the geographical distribution of the reported cases in the Philippines, and annual rice production per province. Although the number of cases in Metro Manila is inconsistent with the expected high incidence of melioidosis among rice farmers, the authors suspected that these patients had previous exposure to agricultural areas. In addition, the proximity and access to diagnostic facility could have caused the relatively higher proportion of cases in Manila. All the indigenous cases were diagnosed in tertiary hospitals in Metro Manila, which generally have better-equipped laboratory and diagnostic facilities than most provincial hospitals, and it is likely that many patients are going undiagnosed outside the city.

The most common co-morbidity was diabetes mellitus, comprising 58.5% of cases, followed by cardiovascular disease (which includes hypertension and coronary artery disease) at 26.82%. Diabetes mellitus is the most common co-morbidity and risk factor found in the literature [16,38,39]. With the steadily increasing prevalence of type 2 diabetes in the Philippines [40], it is likely that the incidence of melioidosis will increase correspondingly.

Pneumonia and soft tissue infection were present in 53.7% and 29.3% of cases respectively, which again is consistent with the melioidosis literature from elsewhere [17]. Pulmonary diseases, particularly pneumonia, chronic lower respiratory tract diseases, and tuberculosis, are among the top 10 causes of mortality in the Philippines [41]. Since melioidosis may be difficult to differentiate from other respiratory diseases, it is possible that many cases labelled simply as ‘pneumonia’ or other respiratory infections may actually have melioidosis. A high index of suspicion, and good laboratory support, are necessary to make a specific diagnosis of melioidosis. Furthermore, co-infection with *B. pseudomallei* and *Mycobacterium tuberculosis* was seen in two patients in this review, which presents a further diagnostic and therapeutic challenge to clinicians. Other than pulmonary cases, four neurological cases were reported among indigenous cases. Although, conclusions could not be drawn, due to limited number of cases, this should be subject for further investigation if this type of melioidosis occur more frequently in the country.

All cases were diagnosed by culture studies. Bacteremia was found in 41.5% of cases. The most common antibiotic used were ceftazidime and trimethoprim-sulfamethoxazole for the intensive phase and eradication phase, respectively. Most of the cases received the recommended antibiotic treatment for melioidosis [42] such as ceftazidime (43.59%), meropenem (12.82%), trimethoprim-sulfamethoxazole (53.84%), and amoxicillin-clavulanic acid (5.13%). Those treated with different antibiotics were cases diagnosed earlier than 1990s, and those indigenous cases which followed the result of the antibiotic sensitivity test. The reported recurrence rate among all cases were 4.9%, while the mortality rate was 12.6%. This, however, could be an underestimate due to lack of data and follow-up. 

Failure to consider melioidosis, which requires specific treatment, may be contributing to the continued high mortality from pulmonary diseases in the Philippines. Local clinical guidelines do not include ceftazidime and meropenem as empiric treatment unless classified as high-risk community-acquired pneumonia, healthcare-associated pneumonia, or those at risk for multidrug-resistant organisms. 

## 4. Challenges

The greatest challenges for the Philippines are to raise awareness of melioidosis amongst clinicians and laboratory staff, and to improve the availability of facilities capable of making a laboratory diagnosis of the disease. During our enquiries with colleagues and local institutions about case reports, many of those who responded admitted to being unfamiliar with melioidosis. Also, melioidosis is rarely included in the curriculum of medical schools within the Philippines. It is notable that the majority of the earlier cases in this series were diagnosed in travelers to other countries, albeit only 21 cases occurring over 70 years, implying considerable under-diagnosis of indigenous cases. However, there are some encouraging signs, particularly, the fact that four unpublished reports, comprising 20 indigenous cases seen over a relatively short span of seven years, were identified. This suggests an increasing awareness of the disease among physicians, resulting in improved diagnosis and reporting. However, these were all diagnosed in Metro Manila, which is unlikely to be the area of highest incidence in a country where the environment is predicted to be widely suitable for *B. pseudomallei* [5], and the incidence of melioidosis in more rural areas requires further investigation. Areas such as those in Mindanao, which are a highly rural area, post additional challenge since access to health care facilities is another existing issue. However, this will require the strengthening of medical laboratories in these areas, and education of staff in how to detect and identify *B. pseudomallei*.

Although the most common presentation of *B. pseudomallei* infection is pneumonia, the 2016 community-acquired pneumonia (CAP) guidelines in the Philippines do not even mention melioidosis as a differential diagnosis. There is no national surveillance system for melioidosis, nor is the disease included in the list of notifiable disease in the Philippines [43].

## 5. Conclusions and Recommendations

This report highlights the fact that melioidosis is indeed endemic in the Philippines but is likely to be grossly under-reported. The clinical and epidemiological characteristics of the disease in the Philippines, albeit based on limited data, are consistent with those described in published literature from other countries. Furthermore, the data show that this condition has been successfully diagnosed and treated in the country with increasing frequency over the past few years.

Further studies should be done on the extent and impact of this disease within the country. It is recommended that a program of education of clinicians and laboratory staff about melioidosis should be initiated, ideally starting at medical schools, and especially targeted at rural areas that are likely to have the highest incidence of melioidosis. Furthermore, consideration should be given to making the disease statutorily notifiable. This would provide more accurate information about the clinical characteristics and distribution of the disease within the Philippines. Soil sampling studies should also be undertaken in order to establish the geographic distribution of this microorganism, as well as enable analysis of the population structure. Genotyping of bacterial isolates are in progress in order to determine if indigenous strains were different than those identified elsewhere in Southeast Asia or Australia. Studies should also be undertaken to describe the current knowledge and attitudes of Filipino clinicians regarding melioidosis.

## Figures and Tables

**Table 1:** Clinical characteristics of culture confirmed cases of melioidosis in the Philippines from published case reports.

Year Published	Age	Sex	Place Diagnosed	Co-Morbidities/Risk Factors	Melioidosis Type	Method of Diagnosis	Antibiotics	Outcome	Reference
1948	25	M	No data	No data	Pulmonary	Sputum culture (guinea pig inoculation)	No data	No data	Gutner and Fisher [6]
1957	32	M	USA	No data	Cutaneous/soft tissue	Sputum culture (guinea pig inoculation); tissue (rib and femoral mass) culture	PEN, SD, STR, CLT, CHL	Recurrent	Prevatt and Hunt [10]
1975	54	M	USA	Lung carcinoma	Pulmonary	Sputum and bronchial washing cultures	TET, CHL	Died	Mays and Ricketts [19]
1976	21	M	USA	None	Pulmonary	Sputum culture	PEN, KAN, SXT	Improved	John [20]
1978	23	M	USA	None	Pulmonary	Sputum culture	TET, CHL, KAN, SXT	Improved	Fuller et al. [21]
1985	46	F	Taiwan	Drowning	Pulmonary	Blood culture	CEF, AMK	Improved	Lee et al. [11]
1994	40	M	Canada	Diabetes mellitus	Pulmonary	Sputum culture	CAZ	Improved	Turner et al. [22]
2002	49	M	Philippines	Diabetes mellitus	Disseminated (joint, pulmonary)	Blood culture	IPM, AMC, SXT	Improved	Ereno et al. [14]
2008	64	M	USA	Diabetes mellitus, coronary artery disease, hypertension	Pulmonary, osteomyelitis	Paraspinal abscess culture, PCR, isolate sent to CDC for confirmation	MEM, SXT	Improved	Falade et al. [23]
2009	61	M	USA	Metabolic syndrome	Pulmonary	Blood culture and pleural fluid culture, PCR	IMP, TGC, SXT, DOX	Improved	Duplessis and Maguire [24]
2010	45	F	Philippines	Diabetes mellitus	Pulmonary	Blood culture	TZP, CLI, AZM	Died	Velasco et al. [25]
2011	50	M	Brunei	Diabetes mellitus	Liver, spleen, cellulitis	Blood culture	No Data	Improved	Pande et al. [26] ^1^
2011	41	M	Brunei	Diabetes mellitus	Cellulitis, pulmonary, liver	Blood culture	No Data	Improved	Pande et al. [26] ^1^
2014	62	M	Canada	Diabetes mellitus, Sjogren syndrome, chronic kidney disease, Stevens-Johnson syndrome secondary to amoxicillin, schistosomiasis, warm autoimmune hemolytic anemia	Musculoskeletal	Blood culture and PCR	LVX, MET, DOX, SXT	Improved	Chagla et al. [27]
2015	60	M	South Korea	None	Pulmonary	Tissue (lung) and sputum culture	No data	Improved	Kim et al. [28]
2015	68	M	USA	Diabetes mellitus, coronary artery disease, hypertension, paroxysmal SVT	Splenic	Splenic abscess culture	CAZ, DOX, SXT	Improved	Guo et al. [29]
2016	44	M	Philippines	Diabetes mellitus	Hepatic	Liver abscess culture	MEM, SXT	Improved	San Martin et al. [15]
2016	67	F	USA	Diabetes mellitus, cardiovascular disease, tuberculosis	Mycotic aneurysm	Blood culture, PCR, isolate sent to CDC for confirmation	MEM, SXT	Died	Hemarajata et al. [30]
2017	60	M	USA	Diabetes mellitus, hypertension, dyslipidemia	Fever, loss of appetite, myalgia, weight loss	Blood culture, isolate sent to CDC for confirmation	CAZ, SXT	Improved	Singh and Mahmood [31]
2017	82	M	USA	Hypertension, hyperlipidemia, osteoarthritis	Mycotic aneurysm	Blood culture	CAZ	Improved	Panginnikod et al. [32]
2018	41	M	Japan	Unknown	Pulmonary, septic arthritis (soft tissue)	Blood culture	DOR	Died	Hadano et al. [33]

AMC = amoxicillin-clavulanic acid; AMK = amikacin; AZM = azithromycin; CAZ = ceftazidime; CEF = cephalothin; CHL = chloramphenicol; CLI = clindamycin; CLR = clarithromycin; CLT = chlortetracycline; DOR = doripenem; DOX = doxycycline; FEP = cefepime; IPM = imipenem; KAN = kanamycin; LVX = levofloxacin; MEM = meropenem; MET = metronidazole; PEN = penicillin; SD = sulfadizine; SXT = trimethoprim-sulfamethoxazole; TGC = tigecycline; TZP = piperacillin-tazobactam; CIP = ciprofloxacin. ^1^ These were Filipino cases diagnosed in Brunei. The infection could have been either be acquired from the Philippines or during their stay in Brunei.

**Table 2:** Clinical characteristics of confirmed cases of melioidosis in the Philippines from unpublished case reports and series.

Year Diagnosed	Age	Sex	Co-Morbidities/Risk Factors	Melioidosis Type	Culture Specimen	Antibiotics	Outcome	Reference
2010	54	M	Diabetes mellitus	Pulmonary	Sputum	TZP, CLR	Improved	Masbang [34]
2010	62	M	Diabetes mellitus, cardiovascular disease	Disseminated (cutaneous/soft tissue, pulmonary)	Blood	CAZ, SXT	Improved	Masbang [34]
2010	55	M	Heavy alcohol consumption	Pulmonary, neurologic	Bronchial washing	MEM, CAZ, SXT	Improved	Masbang [34]
2011	66	M	Squamous cell lung carcinoma, hypertension, heavy smoker	Pulmonary	Sputum	MEM, AMC	Improved	Masbang [34]
2011	54	F	Diabetes mellitus, hypertension	Pulmonary	Blood	FEP	Improved	Masbang [34]
2012	60	M	Hypertension	Pulmonary	Sputum	MEM, SXT	Improved	Masbang [34]
2013	45	M	Diabetes mellitus	Neurologic	Sputum	MEM, CAZ, DOX	Improved	Masbang [34]
2014	57	F	Lung adenocarcinoma, hypertension	Pulmonary	Blood	CAZ, LVX	Died	Masbang [34]
2014	38	F	Breast cancer	Pulmonary	Endotracheal aspirate	CAZ	Died	Masbang [34]
2014	64	M	Hypertension	Cutaneous/soft tissue	Abscess	CAZ	No data	Masbang [34]
2014	47	M	Cardiovascular disease	Pulmonary	Sputum	CAZ	No data	Masbang [34]
2014	59	M	Pulmonary tuberculosis	Pulmonary, cutaneous/soft tissue	Blood, sputum, and wound swab	FEP, CLI, MEM, SXT, TB antibiotics (Isoniazid, Rifampicin, Ethambutol, Pyrazinamide)	Improved	Ocampo [35]
2013	40	M	Diabetes mellitus, poultry worker	Disseminated (soft tissue, liver, intra-abdominal infection)	Abscess	CAZ, SXT, DOX	Improved	Yap et al. [36]
2009	34	M	Diabetes mellitus	Pulmonary	Blood	CAZ, SXT	Improved	Santos [37]
2008	36	M	Diabetes mellitus	Hepatic	Abscess	CAZ, SXT	Improved	Santos [37]
2012	59	M	Diabetes mellitus	Disseminated (cutaneous, joint, pulmonary)	Synovial fluid, wound swab	CAZ, SXT, DOX	Improved	Santos [37]
2009, 2012	44	M	Diabetes mellitus	Soft tissue (parotid), neurologic (chronic and recurrent)	Abscess	2009: CAZ, CIP, 2012: CAZ DOX, CHL ^1^	Recurrent	Santos [37]
2009	50	M	Diabetes mellitus, hypertension, coronary artery disease	Pulmonary	Pleural fluid	CAZ, SXT, DOX	Improved	Santos [37]
2011	43	M	Diabetes mellitus, hypertension	Neurologic	Frontal cortex abscess	CAZ, SXT	Improved	Santos [37]
2009	58	M	Diabetes mellitus, hypertension	Urinary tract, CKD	Urine	CAZ, SXT	Improved	Santos [37]

AMC = amoxicillin-clavulanic acid; AMK = amikacin; AZM = azithromycin; CAZ = ceftazidime; CEF = cephalothin; CHL = chloramphenicol; CLI = clindamycin; CLR = clarithromycin; CLT = chlortetracycline; DOR = doripenem; DOX = doxycycline; FEP = cefepime; IPM = imipenem; KAN = kanamycin; LVX = levofloxacin; MEM = meropenem; MET = metronidazole; PEN = penicillin; SD = sulfadizine; SXT = trimethoprim-sulfamethoxazole; TGC = tigecycline; TZP = piperacillin-tazobactam; CIP = ciprofloxacin. ^1^ Case readmitted after three years, due to recurrence of melioidosis.

**Table 3:** Summary of clinical characteristics of culture-confirmed cases of melioidosis in the Philippines.

Clinical Characteristics	Number of Cases
**Mean Age**	50.2
**Sex**	
Male	35 (85.4%)
Female	6 (14.6%)
**Comorbidities**	
Diabetes mellitus	24 (58.5%)
Cardiovascular disease	11 (26.82%)
Cancer	4 (9.76%)
Pulmonary tuberculosis	2 (4.9%)
Chronic kidney disease	1 (2.4%)
Drowning	1 (2.4%)
Heavy alcohol consumption	1 (2.4%)
Hyperlipidemia	1 (2.4%)
Osteoarthritis	1 (2.4%)
**Organs involved**	
Pulmonary	22 (53.7%)
Soft tissue	12 (29.3%)
Hepatic	5 (12.2%)
Neurologic	4 (9.76%)
Splenic	2 (4.9%)
Mycotic aneurysm	2 (4.9%)
Osteomyelitis	1 (2.4%)
**Positive cultures**	
Blood	17 (41.5%)
Respiratory (sputum)	16 (39.0%)
Abscess pus	7 (17.1%)
Wound swab/Tissue	3 (7.3%)
Synovial fluid	1 (2.4%)
**Outcome**	
Improved	22 (53.7%)
Relapsed	2 (4.9%)
Died	6 (14.6%)
No Data	5 (12.2%)
